# Some Practical Remarks on Chronic Rheumatism*Read before the Oxford Medical Society, July 21, 1880.

**Published:** 1880-12

**Authors:** William Pepper

**Affiliations:** Professor of Clinical Medicine in University of Pennsylvania


### SOME PRACTICAL REMARKS ON CHRONIC RHEUMATISM.* By WILLIAM PEPPER, M.D., Professor of Clinical Medicine in University of Pennsylvania.

As the remarks I propose to make to-day are purely of a practical character, I shall be able to avoid all discussion of disputed theoretical points. Nor have I any idea of attempting a description of all the varieties of chronic rheumatism so-called, or a recital of all the innumerable remedies recommended for their

---

* Read before the Oxford Medical Society, July 21, 1880.

treatment. My only object is to try to sketch a few of the groups of cases that come most frequently under my observation, and to mention the practical lessons I have learned from their study.

In the first place, while it is undoubtedly true that some cases of so-called chronic rheumatism are really rheumatic in nature, it is equally certain that others are not so. It seems to me too much the custom to call a case one of chronic rheumatism simply because the seat of the disease is in one or more joints. It is true we are entirely ignorant of the essential nature of rheumatism, but at least all agree in considering that it is a general or constitutional disease, and that the articular affections are merely its local manifestations. On the other hand, it cannot be doubted that the different tissues entering into the formation of a joint are liable to various other sorts of diseased action. Gouty or syphilitic inflammation may occur; traumatic irritation is very common, and simple idiopathic inflammation from ordinary causes, such as damp, changes of weather, etc., may occur here as elsewhere. In addition to this, it would be found on careful examination, I am sure, that many painful affections of tissues near joints are commonly called rheumatic, although the joints proper are themselves not involved, unless secondarily.

Among the cases that may be selected in illustration of these remarks, I have met with a strikingly large number where the shoulder joints have been affected. The symptoms with which such patients have presented themselves have been pain referred to the shoulder joint, and inability to make the ordinary movements of the arm, particularly to raise it above the head or to put the hand behind the back.

In the majority of cases only one shoulder is affected, but I have met with a good many instances where both were involved. Sometimes the affection has been of a rheumatic character, and either originally formed part of a general articular disease, or was from the first the only local manifestation of a constitutional disturbance. But frequently no such rheumatic element could be assumed positively; but the affection appeared purely a local one. The cause has occasionally been traumatic—as a fall, striking on the open outstretched hand, or so as to drive the head of the humerus violently against the glenoid cavity, or so as to put on the stretch and irritate the synovial capsule and the nerves which pass in close proximity to the joint. The same result has been caused by a blow on the shoulder, or by a sudden and violent muscular exertion. In other cases, a sudden chilling of the surface while overheated, as by a cool draught blowing on the shoulder, has excited the inflammation. Sometimes the trouble has come on very insidiously, by the repetition of trifling and almost unnoticed irritations, until finally a state of positive disease is established. But, however excited, the anatomical conditions and the symptoms are similar.

Pain is, as I have said, a constant symptom. It is often worse at night, and interferes much with sleep. It is of a wearing, sickening character, and is increased by attempts at motion or by allowing the arm to hang downward. The head of the humerus quickly assumes the position of a slight subluxation forward and downward. Pressure with the finger along the course of the brachial plexus constantly reveals decided local tenderness of the nerve trunks, due to a neuritis or perineuritis that has resulted either from the original cause of the attack, or from an extension of inflammation from the synovial capsule. In case the head of the humerus is allowed to occupy its unnatural position, the irritation of the nerve trunks is greatly increased by the pressure of the head of the bone, and the secondary neuritis becomes more serious and extensive. The circumflex and the median nerves are those most commonly involved. The pain now radiates along the course of these nerves and especially extends down the arms and into the fingers; a feeling of numbness and tingling, or burning, is apt to accompany it. Liquid effusion into the synovial capsule is rare, but a tendency to adhesive inflammation rapidly shows itself, and in a wonderfully short time slight, false anchylosis develops, which, if neglected, grows more and more close and firm, holding the head of the humerus with constantly increasing force in its abnormal position. From the very first, the power of movement of the arm is much impaired. The hand and forearm do not share in this; but the arm can be lifted only a short distance from the side, so that the hand cannot be fully elevated. Rotation of the humerus is prevented so that the hand cannot be carried behind the back. The patient soon finds he cannot use the hand on the affected side either in eating or in dressing.

The angle to which the arm can be raised varies greatly in different cases, and in testing the power of motion it is necessary to guard against error by fixing the scapula by firm pressure, since otherwise the patient will unconsciously deceive by tilting the thorax toward the sound side, and thus apparently bringing the humerus on the affected side to a higher level.

The cause of this impaired mobility is at first the instinctive avoidance of pain and the lessened power of the deltoid from the irritation of the circumflex nerve, which is distributed to this muscle. But other influences soon come into play. Adhesions form and hold the head of the bone more and more firmly; the deltoid soon begins to waste from disuse, and the inflammation of the circumflex nerve impairs the nutrition of the muscle and hastens its atrophy. The dependent position and disuse of the arm, and the interruption of circulation caused by the pressure on the veins, lead to passive congestion and œdema of the hand and forearm. If a descending neuritis is established in the ulnar and median nerves, atrophy of the muscles of the forearm and hand ensues ere long, and, finally, a most helpless condition of the member is brought about.

Brief notes of a few illustrative cases may be interesting:

CASE 1.—Mr. S. R. S., æt. 50, banker, fell on ice, and holding out left hand to save himself, felt a sharp pain in left shoulder joint. When seen, two days later, there was inability to lift left arm more than to angle of 45° from body, and that was very painful. There was exquisite tenderness over the brachial plexus; the circumflex nerve felt swollen, and was especially painful. The arm was supported so as to carry upward and backward the head of the bone. Passive motion was begun at once, and continued daily so as to prevent adhesion; a blister was applied over the inflamed nerve trunks; iodide of potassium and bichloride of mercury given internally. In a few days the soreness was greatly relieved, and then Faradic electricity was applied daily to deltoid; more thorough passive movements were practiced, and a very rapid cure followed.

CASE 2.—George Kiegel, æt. 57, carter, came to me on April

24, 1877. The month previously began to notice pain in right shoulder ; shortly before that had fallen on the ice. The aching pain continued, and in two weeks he had to give up work, and in two weeks more he could not dress himself. The case had been regarded as one of rheumatism. The arm had been kept quiet, and internal and local remedies used. The pain and helplessness steadily increased. When I first saw him, he could not lift humerus from side at all. The deltoid was markedly atrophied. The head of the humerus was in advance of its normal position, and was very firmly held there by strong adhesions. There was intense tenderness with marked swelling of the nerve trunks in front of the head of the humerus. There was severe pain about the shoulder, also extending down the arm along the course of the nerves, most acutely felt at elbow and at interphalangeal joints. This latter pain was much increased by closing the hand. There was numbness of hand and arm, and occasionally the hand became swollen. Unquestionably storms and sudden changes of weather increased pain, numbness and weakness. With dynamometer right hand gave only 40; left hand 110. Iodide of patassium, gr. v, t. d.; repeated blistering over the inflamed nerve trunks; persistent graduated passive movements until adhesions were all stretched and broken, and then faradization of deltoid and shoulder group of muscles, constituted the treatment. By June 12, 1877 (seven weeks), the arm could be moved passively in all directions; the deltoid was regaining its size and power, and power of movement had returned to a great extent, although he was not yet allowed to work. R. hand with dynamometer 100. Pain entirely relieved.

CASE 3.—Mrs. M., from Chester, Penn., æt. 45, accustomed to doing rather laborious housework, applied to me in 1875 for the relief of extreme pain in the right arm, combined with total helplessness. She had evidently taken cold repeatedly while overheated, and had had repeated slight attacks of pain about the right shoulder joint. Finally the pain grew so severe that she was obliged to give up work, and soon she found herself unable to lift her arm from her side, or lift her hand to her head to feed herself, or to use it in dressing herself; and finally it grew almost entirely useless. During this time she suffered constant pain.

It was of a wearing, dull character, in the shoulder joint, frequently shooting down the arm to the fingers, and was so severe at night that she scarcely slept at all; and her general health had suffered greatly, with much loss of flesh in consequence. The condition had lasted for several months when she first consulted me. She stated that she had been treated for chronic rheumatism, and that she had been recommended to keep the arm at rest. There was advanced atrophy of the right deltoid muscle, and the application of the faradic current, or of a slowly interrupted galvanic current, caused scarcely any contraction of its fibers. The head of the right humerus was firmly fixed in a position of slight subluxation forward and downward, and any attempt to rotate it, or to elevate it in any direction, met with firm resistance, and caused intense pain. The cords of the brachial plexus were swollen, hardened, and exquisitely tender. The muscles of the right arm and forearm were somewhat atrophied; the hand was puffy and swollen, and there were severe complaints of burning and tingling pain, with numbness down the arm and through the hand. Systematic manipulation of the arm, directed toward breaking up the adhesions, was used at intervals of about five days, despite the intense suffering caused. After each treatment, however, the pain was lessened, and mobility was increased.

She was also exhorted to use the arm as much as possible, carrying her efforts as far as her endurance would enable her to do. Repeated blisters along the inflamed nerves were used; the whole shoulder was enveloped in a batt of wool saturated in a strong liniment of chloroform and aconite. Iodide of potassium and bichloride of mercury were given internally. After some degree of mobility was restored, massage of the deltoid, with occasional faradization, was used.

Electromotor contractility gradually returned, and the muscle gained in bulk satisfactorily. Treatment extended over three months, by the end of which time pain was entirely relieved, and she was able to use her arm quite freely. She was directed subsequently to continue regular gymnastic exercise with it, so as to thoroughly complete the restoration of motion and power, and

I learn now (June, 1880), that the arm has long since returned to its normal state.

I could quote from my case-books the records of a very large number of instances presenting the same essential conditions as the last; but I will only tax your patience by reading the notes of one of a different type, although illustrating some of the same points.

CASE 4.—Mrs. R. A., sent to me Sept. 4, 1875, from Belmont county, Ohio. She was about 40 years of age, and had enjoyed general good health. Two years previously, numbness in both hands came on quite suddenly, and gradually grew worse, extending up arms to shoulders. There was gradually increasing weakness of arms, and frequent aching pain, especially before changes in the weather, in shoulders and down arms. The case had been regarded as one of chronic rheumatism; she had been directed to keep the arms as quiet as possible; liniments had been used and anti-rheumatic remedies given internally. The left arm was the worse. The deltoid was considerably atrophied, and quite close anchylosis of the shoulder joint existed. The muscles of both arms, especially the left, were decidedly atrophied.

Unquestionably the beginning of this interesting case was a rheumatic neuritis of the nerve trunks (medians or ulnars) in both arms, which ascended until it reached the circumflex nerve and the brachial plexus. Partial loss of power of the deltoids (particularly the left) had combined, with intentional disuse, to allow the head of the humerus to remain comparatively motionless, until adhesions formed between it and the glenoid cavity.

Passive movements until all adhesions were broken up; regulated massage and exercise; persistent counter-irritation along course of inflamed nerves; the use of the constant galvanic current, and the internal use of iodide of potassium with small doses of bichloride of mercury, was the treatment directed. The patient returned home at once, and the result is unknown.

It is not only in reference to affections of the shoulder joint that a careful study of the adjacent nerve trunks is important, but I know no other joint where arthritis is so apt to be associated with neuritis. Sometimes the neuritis is the primary trouble, and the joint becomes involved secondarily; more commonly, the arthritis precedes and a secondary neuritis from extension of irritation ensues. The essential point, however, is to recognize the two elements and to adapt the treatment accordingly. If the case be seen at an early stage, before any anchylosis or atrophy of the deltoid has resulted, a rapid cure can be effected by the use of a suitable bandage to support the arm and carry the head of the humerus upward and backward, thus obviating any pressure on the nerves or vessels; by active counter-irritation along the course of the nerve trunks if they are found tender and swollen; by the internal use of full doses of quinia, together with iodide of potassium and bichloride of mercury; and, as soon as the acute inflammation is subdued, by the application of a galvanic current, the positive pole being placed over the affected nerves and the negative pole over the deltoid muscle.* But while these measures are being carried out, it is essential that as soon as the acute stage has passed by (say after the first two or three days), gentle and gradually increased passive movements of the arm should be practiced.

But, in my own experience, such cases have much more commonly come under observation at a later stage, and when more or less serious changes have occurred. The first point of importance, then, is the diagnosis, and there are several conditions with which it is possible to confound the affection we are considering. In the first place, finding the head of the humerus somewhat displaced from its normal position, the shoulder decidedly flattened, and the movements of the arm much restricted and painful, and learning possibly that some fall or twist had preceded the trouble, the idea would naturally occur of a subluxation of the humerus. Indeed, as I have already said, there does come to be a slight degree of subluxation, and in very chronic cases where immobility has been allowed to continue for a long time, the glenoid cavity undergoes such changes as to render it impossible for the head of the humerus ever to resume its normal position. But I have known cases where, after the acute stage had been injudiciously

* We owe to Remak the demonstration of the great value of the constant current thus applied in cases of articular neuritis, accompanied with paresis of the deltoid. See his noteworthy "application du courant constant au traitement des névroses," Paris, 1865, pp. 41. Extracted from *Revue des Cours Scientifiques*.

treated with the usual result of anchylosis, the patient has fallen into the hands of unscrupulous charlatans or ignorant bone-setters, who have been shrewd enough, however, to recognize the necessity of forcible motion of the joint, and then, on finding, after one or two sharp cracking sounds have been distinctly perceived, that the bone returns more nearly to its normal position, and that marked improvement in the power of movement has ensued, have advanced the theory that the case has been one of neglected subluxation from the first, and that damages for malpractice should be claimed. Such an error would be impossible at the early stage of the case; and later, by careful attention to the history and evolution of the case, and by observing that the restriction of mobility is not only in the direction that would result from a subluxation forward, and that just in proportion as the adhesions are broken up by gradual passive exercise, normal mobility returns, it is possible to avoid any mistake.

I have repeatedly known such cases to be regarded merely as paralysis with atrophy of the deltoid, and a treatment of persistent faradization, hypodermic injections of strychnia, etc., to be carried out, but, of course, without any result, because the cardinal fact was overlooked that the paralysis and atrophy of the deltoid (which undoubtedly existed) resulted from: 1st, neuritis of the circumflex nerve, excited and maintained by the articular trouble and the abnormal position of the humerus; and 2d, by disuse owing to neglected anchylosis of the shoulder joint. The mode of development of the case, the early impairment of motion in directions not requiring the action of the deltoid, the pain and tenderness, and, finally, the anchylosis—all render easy the recognition of the true nature of the case, and show that the conditions of the deltoid are purely secondary.

The mode of treatment that succeeds, even in very bad and long continued cases, has been, perhaps, sufficiently alluded to in the brief records of several cases already given. A few words may be added, however, in regard to some of the points.

Systematic passive movement and massage are the most essential parts of the treatment. Without these to free the head of the bone from its abnormal position and relieve pressure on the nerves and vessels, all else must fail utterly; while just in proportion as the adhesions are broken up, all other symptoms improve. I may say that, in my experience, etherization and the forcible breaking of the adhesions has never resulted as favorably as their more gradual destruction by repeated, comparatively gentle passive movements. Used with the utmost care, however, great suffering is always caused; but this must be disregarded, and the manipulations be steadily persisted in, since all delays increase the atrophy of the muscles and render a cure less hopeful. In addition, the patient should be encouraged to use the arm as much as possible, instead of being allowed or directed to keep it quiet.

Pain is often intense. I have frequently known the general health suffer gravely from the interference with sleep. The removal of the adhesions is the surest mode of affording relief, but more immediate methods are needed. The hypodermic injection of morphia and atropia, the application of strong veratria ointment, or of a strong liniment of aconite and chloroform, and the application of the constant galvanic current are the most prompt and reliable remedies.

Neuritis is so common a complication that the condition of the nerve trunks (especially the circumflex and the median and ulnar) nearly always calls for treatment. Repeated small blisters along the course of these nerves, and the use of the galvanic current in the way already mentioned, i. e., with the positive pole over the irritated nerve trunk, and the negative pole over the fibers of the deltoid, is the best treatment, combined with the internal use of iodide of potassium with small doses of mercury. I should not limit the use of these remedies to those cases only where a rheumatic element clearly exists, but would recommend their use generally for the relief of the chronic arthritis and of the inflammatory swelling of the nerve trunks.

As a further illustration of an analogous form of articular and neural trouble, although involving a different member, the following interesting case may be cited:

CASE 5.—Mr. D., of Mahanoy City, age 38, has never had syphilis, but has been much exposed to wet and cold. In August, 1877, began to suffer with pain and swelling of the right knee, and with pain in the muscles of the back, and extending thence around the limb, and down the front of the right thigh as far as the knee. On August 18th the pain became very violent, and he was confined to bed for five weeks; since then it has not been so violent, though subject to frequent severe, sudden shocks. There has also been constant pain about the right knee and along the thigh. He consulted me November 1, 1877. The right knee joint was swollen, with some effusion, painful and stiff. The anterior group of thigh muscles were weak and decidedly wasted. Marked tenderness existed along the course of the anterior crural nerve. The urine presented a heavy deposit of urates. A plaster of ammoniac and mercury was applied over the knee; blisters were applied along the course of the affected nerve; mild faradization of the muscles of the thigh was employed daily; and internally iodide of potassium, three grains, increased to six grains, with Donovan's solution, ten drops, thrice daily, were given. In the course of two weeks improvement was marked, and went on steadily to complete recovery. When last seen, in September, 1879, he remained perfectly well.

I have dwelt thus minutely on this form of articular trouble, not only because I am led to believe it is a rather frequent affection, and one which does not always receive prompt recognition and appropriate treatment, but because it seems to me to illustrate clearly the important points: that painful articular affections are by no means always of a rheumatic character; that many of the symptoms connected with articular affections may be due to implication of surrounding tissues, and particularly to inflammation of adjacent nerve-trunks; that the paralysis and atrophy of muscles connected with the affected part, which constitute most serious complications, are often attributable to the influence of a neuritis, more than to that of prolonged inaction; that in many cases of arthritis without liquid effusion there is a strong tendency to the formation of adhesions; and that this false anchylosis, particularly where, as in the shoulder joint, the bone becomes fixed in an abnormal position, constitutes a serious complication and should be prevented by an early resort to gentle, systematic passive movements.

In connection with this most important and difficult question of when to use rest and when to use movement in the treatment of articular affections, I will allude briefly to the subject of rheumatoid arthritis. It is clear that this interesting disease has no direct relations with either acute articular rheumatism or with true gout. But it has unmistakable analogies with conditions that must be grouped under the name of chronic rheumatism. It attacks persons who, whether from inherited tendencies or from acquired weakness, present conditions of depressed vitality with abnormal sensitiveness to the action of damp and climatic changes. In my own experience, by far the most common and demonstrable cause has been residence in a damp house or a damp locality, operating on a system enfeebled by such depressing influences as excessive child-bearing and sexual exhaustion, or as a severe prostrating illness, such as typhoid-pneumonia. There seems to be developed gradually, perhaps in consequence of the defective action of skin caused by the prolonged action of damp or by repeated chillings of the surface, a morbid state of the synovial tissues, and to a greater or less degree of the subjacent articular cartilages. One joint after another becomes involved, with a certain regard to symmetry, but without regard to the size or locality of the joints. Some effusion occurs at first into the synovial capsules, but later this is apt to be absorbed; the synovial membrane is thickened and roughened; in places destruction occurs both of the membrane and of the subjacent articular cartilage. Meanwhile the margins of the joints are involved, and ridges or nodules of new-formed bony tissue appear; the fibrous tissues become thickened, and the tendons no longer play freely through their sheaths; the whole joint becomes more and more distorted and useless; motion grows more and more painful and difficult; finally, firm anchylosis occurs with great deformity, and the functions of the joints are utterly destroyed. One point of very great practical importance in connection with these cases has not been sufficiently noticed. It is the fact that the subacute inflammation extends from the surrounding fibrous tissues to the sheaths of the nerves, and an ascending and descending neuritis is apt to be set up. Not only does this complication cause a serious addition to the suffering in the form of pain radiating along the course of the affected nerves, but it induces grave nutritive changes, such as a more rapid and extreme atrophy of the muscles than would result from mere disuse, and even degenerative lesions of the skin and nails. The terrible state of helplessness to which the unhappy victims of rheumatoid arthritis are brought, in the later stages of the malady, is familiar to you all, and is well illustrated by this sketch of a patient now in the ward of Phildelphia Hospital, for which I have to thank the skillful brush of my friend Dr. J. M. Taylor.

It is needless to say that by the time a patient reaches any such condition as this, he is far past all hope of real relief from medical treatment. But in the earlier stages I have had numerous occasions of late years of seeing what vast improvement can be effected by systematic treatment of a certain character. I began the study of rheumatoid arthritis with the idea that it was a hopelessly incurable disease. I was aware that every remedy in the pharmacopœia had been used without positive benefit in its treatment. I can add my testimony, after prolonged and faithful trials, that little is to be expected from any of the well-known anti-rheumatic remedies in rheumatoid arthritis. And yet I have become satisfied that in many cases—and I mean to include severe cases and quite advanced stages of the disease—vast relief can be afforded to the symptoms, the progress of the disease can be checked, and even a considerable degree of usefulness be restored to badly crippled joints.

The notes of the two following cases may be cited here as illustrative, both of the symptoms of rheumatoid arthritis and of the general features of the plan of treatment to which I beg to call your attention:

CASE 6.—Mrs. C., living near Woodbury, N. J., came under my care in March, 1878. There had been no rheumatism or gout among her grandparents, uncles or aunts. Her father died of palsy at seventy-two, her mother of puerperal fever at thirty-nine. She had herself one sister who began to have arthritis at three years of age, and ended by having her joints greatly distorted; finally the disease became inactive, and she lived to the age of thirty-eight years, when she died in childbirth. Mrs. C. herself always enjoyed good health. She was always very sensitive to changes of weather, and required a great deal of clothing. She ceased menstruating at the age of forty-nine. About the same time she was subject to great mental distress. While passing through the menopause she noticed subacute inflammation of the right great toe, which soon spread to the joints of other toes, and then invaded the wrists and hands, then the knees, then the hips, and at last extended to the elbows and shoulders; the ankles alone were never much affected. The pain in the affected joints was very violent, especially at night; it was apparently influenced by atmospheric changes. It was chiefly seated in the affected joints, but there were also at times lancinating pains along the members. There was marked wasting, with great loss of power of the muscles connected with the affected joints.

There was marked deformity of many of the joints, of the character typical of rheumatoid arthritis. She did not suffer with headache and there was no spinal tenderness, but there was pain in the lower part of the spine. She was confined to a rolling chair. She could move her arms at the shoulders pretty well, but the elbows were very stiff and the wrist rigidly fixed. The legs were fixed at a right angle so firmly that great force was required to elevate them, and motion caused extreme pain. There was marked wasting of the extensor muscles of the thighs, with great tenderness on pressure along the course of the nerves and at certain points along the shafts of the femurs. There was no cardiac disease.

The patient was removed to the University Hospital, where she remained for thirty days, and then returned to her home in Woodbury. Systematic manipulation of the affected members was practiced daily, with forcible movements of the stiffened joints. This movement was effected partly by skillful massage, partly by various mechanical appliances. She was especially urged to make regular muscular exertions herself, and to use the appliances provided to effect as much motion of the joint as possible. A considerable portion of every day was consumed in this regular gymnastic work. At first very limited motion only was possible, but gradually all the joints except the wrists yielded and became much more movable. Regular daily frictions of the whole surface with oil, and twice weekly with alcohol, were also used; extreme care in dressing was urged, as also in regard to exposure to draughts or to sudden changes of temperature.

Thorough treatment by faradization of the whole muscular system was carried out on alternate days. Internally she took nitrate of silver and small doses of opium and belladonna, given in pill form, thrice daily, until 30 grs. of the silver salt had been taken; and, after an interval of three weeks, another course of 20 grs. was given. Dialyzed iron in doses increasing up to one fluid drachm, thrice daily, was given steadily for a long time. In the intervals between the courses of nitrate of silver she took iodized cod liver oil.

When she left the hospital she was acquiring gradually increasing power of motion in her joints, and the wasted muscles were gaining in size and strength. She had begun to walk with but slight assistance. Subsequently the same line of treatment was carried out with most gratifying results. The pain was almost entirely relieved, her general health became much better, and at the last time I heard from her she was able to move her joints even more freely than at any previous time of her sickness.

I will also give a brief abstract of the notes of the following interesting case, because it was an example of great benefit obtained in an apparently hopeless state (and because the patient may perhaps be well known to some of my hearers).

CASE 7.—Mrs. S., of Wakefield, Lancaster Co., Penn., lived in a house which was probably quite damp, and at the age of twenty-seven, while feeling somewhat run down, she had a severe attack of pneumonia of the left lung, ten days after which she was taken with inflammation of the left shoulder and elbow, soon followed by inflammation of both knees and hips. The pain was very acute even during entire rest, especially so at night, and was much increased by motion. Since then the disease had progressed with acute exacerbations at irregular intervals, at which time new joints became affected, and those already diseased became worse. In the intervals the acute symptoms would subside to some degree. At the time of the exacerbations there was usually some fever. The urine had frequently shown deposits of urates. The general health suffered severely; there was great loss of flesh, color and strength; sleep became very poor, being disturbed by pain, and also by painful contractions of the flexors of the lower extremities. During several months previous to my first seeing her, these contractions were especially severe. There was tenderness over one point of the lumbar spine, but no evidence of spinal disease. The habit of using morphia freely had existed for a considerable time. On admission to the University Hospital, October 23, 1877, the disease having lasted six years, Mrs. S. was almost entirely helpless, at the age of thirty-four. She was confined to her chair, being entirely unable to straighten herself or to stand. There were stiffness and impaired motion of the cervical spine. There were excessive pain and tenderness of the affected joints, all of which presented the most characteristic changes and deformities of rheumatoid arthritis. These consisted of swelling and deformity; within some a moderate amount of effusion in the synovial sacs, and in others stiffness or firm anchylosis; lesions of the synovial membrane and articular cartilages as shown by crackling and crepitus on motion; and, finally, new bony formations around the margins of the affected joints. There was advanced wasting of the muscles nearly all over the body. An imperfect response was given to faradic currents. Pain was extreme, and although her endurance was heroic, the nervous system was considerably disturbed. There were extreme pallor and marked emaciation, the weight being only 104 pounds. Not only were the legs immovably fixed, but the arms were likewise crippled so that she could not feed herself; and the hands were entirely useless. The contractions of the legs, already noted, were very marked and painful. They came upon her frequently and without any cause, although effort would always provoke them. Owing to the stiffness of the knee-joints they did not cause very much drawing of the legs upward, but the flexor muscles could be seen or felt to contract in a sudden spasmodic manner, and then to soon relax more slowly and irregularly. In addition to these large muscular contractions, sudden jerking movements of the fingers were noticed. Even those which were dislocated in consequence of the articular changes, would be suddenly seized with jerking, and would shake rapidly and uncontrollably. Fibrillar contractions of the muscles of the hands and of the forearms had been noticed for a year past.

Her diet was very carefully regulated, and she was encouraged to take largely of light nourishing food, in addition to considerable amounts of milk.

A pill of nitrate of silver, gr. $\frac{1}{4}$, extract of opium gr. $\frac{1}{4}$, extract of belladonna gr. $\frac{1}{8}$, t. d. p. c., was ordered. Daily inunctions of oil, with massage of the whole muscular system, and systematic manipulation of all the affected joints, were faithfully kept up.

Mechanical appliances were devised to gradually break up the adhesions of the larger joints, and arrangements were made by which, as soon as a little movement in any joint was secured, systematic exercise of the muscles attached, could be maintained. The muscles were carefully and gently faradized.

In the course of a month there had been considerable improvement. The appetite was better, and she had commenced to gain much flesh. There was less tendency to exacerbations, and her pain was diminished, so that she could sleep fairly well without morphia and with diminishing doses of opium in her pills. The joints had yielded to manipulation better than seemed possible, so that movement was returning in many of them, and their enlargement was diminishing. The muscles responded better to electricity. Spasmodic contractions of the legs at night continued, but this was relieved by bromide of potassium. She began to take dialyzed iron about December 1st, and took it for many months; it was impossible, however, to give it to her in larger doses than fifteen to twenty drops, thrice daily, for when increased beyond this it caused looseness of the bowels. She took 40 grains of nitrate of silver continuously, then stopped it for several weeks, and resumed it, taking 15 grains more; and this she repeated until she had taken in all about 75 grains while at the hospital. Her improvement was slow but steady; by February 1st her weight had increased to 114 pounds. Marked improvement had occurred in the power of motion, and in the anatomical condition of the joints. She became able to help herself in many ways, and finally to walk about with the assistance of the Darrach wheeled crutch. She left the hospital in the summer of '78, to return to her home, where the same treatment was to be carried out.

By December 15, 1879, her weight had increased to 140 pounds—a gain of fully 36 pounds since she first came under my care.

She was almost free from pain; her functions were all well performed, and there was great improvement in the mobility and power of motion of nearly all the joints. She was able to walk considerably with the apparatus named, and could also run a Howe's sewing machine herself. Massage and inunctions had been steadily continued, and faradization had been used occasionally.

She had been taking dialyzed iron constantly for over two years, and since leaving the hospital had used 75 grains of the nitrate of silver in the course of eighteen months, making 150 grains in all. While some of the joints remained anchylosed, or distorted, in consequence of the advanced lesions that had been developed previous to her coming under my care, it may safely be said that the improvement in this interesting case was most gratifying and encouraging.

In these cases, and so in all similar cases, where I have succeeded in effecting any material relief, one of the most important, or probably the most important element in the treatment has been systematic daily manipulation. This includes persistent movement of all the affected joints, excepting those where anchylosis has been allowed to become so firm that any motion is impossible. But even when complete immobility has apparently been established, I have frequently been surprised to find that vigorous efforts have restored some measure of usefulness to the part. In the case of joints where the inflammation presents a very acute stage, attended with rapid swelling and decided heat and redness, it is proper to await the subsidence of this severe irritation before instituting regular manipulations; but the delay need rarely be long. Of course such manipulations are excessively painful, and must be conducted daily without anæsthesia. Still, so highly important do I consider this treatment, and so excellent are the results often obtainable by it, and by it alone, in cases which otherwise would pass steadily into more and more settled helplessness, that I feel no hesitation in appealing, and always with success, to the patient to undergo it thoroughly and persistently. Where the tendency to contraction, deformity and anchylosis is too great to be overcome by ordinary manipulation, suitable apparatus may be contrived to assist in overcoming it, as was done in Case 7.

I would beg to repeat, then, that in rheumatoid arthritis, from an early to a late stage, despite the pain occasioned by such manipulation, I consider the most essential part of treatment to be the systematic daily movement of the affected joints, combined with thorough massage of all the muscles whose functional activity is impeded and impaired. It is not, indeed, alone the maintenance of the mobility of the joints that is arrived at in such treatment. the circulation of the tissues around the joints is stimulated, and the tendency to absorption of the exudation is increased. The nutrition of the muscles is maintained, and the atrophy of their tissue, that we have all had occasion to note as among the most serious results of this form of joint disease, is as far as possible obviated. But in addition to all this, there seems every reason to believe that in this affection, as in nearly all cases of chronic rheumatism also, there is an underlying impairment of the tone and activity of the skin which is the strongest predisposing cause. There is no way in which this can be improved so well as by systematic manipulations and frictions, accompanied by the use of suitable baths, or by inunction with a vegetable oil. In feeble, anæmic rheumatic patients, where even hot salt baths of very short duration may be badly borne, or, on account of the crippled joints, may be inconvenient, the thorough daily inunction of the whole surface with pure olive or cocoa oil has for a number of years been a favorite practice with me.

In many such cases change of residence, and, if possible, change of climate is extremely beneficial. The diet nearly always requires close attention, and, as a rule, it is necessary to arrange it so that a much larger quantity than formerly shall be taken of simple, wholesome food, which is often best done by adding two or three pints of skimmed milk per diem to the regular diet.

It has long been recognized that patients with rheumatoid arthritis usually present an anæmic condition, and that nutrients and alterative tonics produce better results than any specific remedies, such as iodine, iodide of potassium, or guaiacum, etc. Of all tonics, iron in very large doses has proved by far the most valuable in such cases, and in unusually large amounts it forms an almost invariable part of my treatment of rheumatoid arthritis.

In many instances, especially where there has been marked pain extending along the nerve trunks, and perhaps associated, as often happens, with considerable disturbance of the nervous system, the prolonged use of nitrate of silver, with or without minute doses of opium and belladonna, has seemed to exert a favorable alterative effect. There is a constant temptation to resort to the anodyne use of opium in some form, but it need scarcely be said that this should be resisted most uncompromisingly, since there is scarcely any disease in which the opium habit is more readily acquired, more injurious in its effects and more difficult to break off. Local applications (veratria, aconitia, chloroform) or counter-irritation (iodine, small blisters, various mechanical irritants, or, finally, the constant galvanic current) may afford relief to pain.

It is not, of course, intended to say that other drugs are not called for in many cases. Cod liver oil and arsenic may supplant iron in cases where the latter constipates too persistently. Iodide of potassium with minute dose of bichloride of mercury, may replace or alternate with the use of nitrate of silver. Long continued courses of lithia, as a substitute for all drugs of this latter class, have proved serviceable, especially in cases attended with acid dyspepsia and the uric acid diathesis.

Electricity has been alluded to as a means of relieving local pain, but its systematic use enters as an essential part into the treatment of every case—not only for its effect on the muscles, but on the superficial circulation over affected joints, and on irritated or inflamed nerve trunks.

Lastly, a most rigid attention to hygiene is essential, including dress, exercise, avoidance of draughts and damp, etc.

This very hasty and imperfect sketch shows clearly enough the well-known truth, that it is not on any one drug or combination of drugs that we are to rely in the treatment of rheumatoid arthritis any more than in other chronic diseases; but that it is only by a thoroughly organized systematic plan of treatment, including hygiene, gymnastics, dietetics and therapeutics, that any success can be obtained. I would beg to emphasize the leading indications in ordinary cases of rheumatoid arthritis, as follows:

To remove the cause—having special regard to residence, soil, moisture, etc.

To maintain at all hazards the mobility of the joints.

To exercise the muscular system.

To restore and maintain the tone of the skin.

To improve the blood and nutrition.

To quiet the pain as far as possible by local means.

To modify the articular inflammation (and that of the adjacent nerve trunks when it exists) by counter-irritation, electricity, and the internal use of alteratives.

It may well be questioned whether such treatment can be successfully carried out under ordinary conditions of home life; and it cannot be doubted but that, for the maintenance of perfect regularity and system in each detail, as well as on account of the skilled attendants and special appliances required, it is generally preferable that such patients should be treated at a suitable institution.

I have spent so much time on the subject of rheumatoid arthritis, that it is not possible to more than glance at some additional practical points in regard to ordinary chronic rheumatism. In the first place, it is clear that in many cases of articular trouble, systematic motion would be injurious, and absolute rest must be enjoined. Here it seems to me better, not merely to allow the patient to lie in bed, trusting that the limb may be kept comparatively quiet, but to apply either a plaster bandage or a carefully adjusted splint, so as to secure absolute rest conjoined with carefully graduated pressure. The cases that call particularly for this complete rest seem chiefly to be those where the arthritis is very acute and painful, and older cases where there is a considerable amount of liquid effusion in the synovial sac. In the latter cases, no danger of anchylosis exists, and, moreover, the distended and weakened synovial membrane is irritated anew by any strong manipulation or extended movements. In fact, I should be inclined to say, that just in proportion as liquid effusion exists in a diseased joint, is passive movement or active exercise undesirable; while in proportion as the joint is free from such effusion, and presents, instead, thickening, stiffness or adhesions, is manipulation (of course, carefully graduated by the activity of the inflammatory process and the sensitiveness of the part) advisable.

As illustrating the excellent results obtained in chronic rheumatic synovitis and arthritis from rest and pressure, combined with nutrient and alterative treatment, the following case may be cited:

CASE 9.—Mr. S., æt. 65, farmer, from Susquehanna county, Penn., was admitted to the University Hospital in 1878. In consequence of working in a damp district, with constant exposure to hard toil, he became gradually crippled with chronic rheumatic inflammation of both knee joints. The shoulders were also affected, but to a less degree. After trying numerous modes of treatment at home and at neighboring mineral springs during the course of several years, and finding that he was growing gradually worse, he went to the Hot Springs of Arkansas, where he used water and baths faithfully, but steadily grew worse, so that he was entirely confined to his bed for several months before being brought to the University Hospital. He was greatly emaciated, anæmic and feeble. The appetite was poor and digestion torpid. He was exquisitely sensitive to the least changes of weather, to draughts and damp, so that in every way he presented the highest degree of atony of skin and general system. The shoulder joints were stiff and painful on movement, but there was no liquid effusion therein. Both knee joints were enormously distended with liquid, so that the legs were in slightly flexed condition, and the least attempt at motion caused extreme pain. The synovial membrane was thickened and crackled when moved, and there was some infiltration of the surrounding tissues. The muscles were wasted and flabby. It was impossible for him to stand for a moment even with help of two canes or crutches.

He was kept in bed strictly. The knee joints were enveloped with plaster bandages, which were changed as frequently as the diminishing size of the joints rendered them at all loose. Manipulation of the shoulder joints and thorough massage of the whole surface and muscular system, with inunction, were employed daily. A carefully regulated diet was directed, and he was encouraged to take very full amount of simple, wholesome food. At first he took quinia, strychnia, and muriatic acid, but as soon as the tone of his digestion improved, he was put on very large doses of dialyzed iron, with full doses of Donovan's solution, and later of KI. and $Hg.Cl._2$. Very gratifying results followed this treatment. He quickly regained use of the shoulder joints. The effusion in the knee joints steadily subsided, and in the course of three months was so far gone that passive movements were well tolerated. He gained twenty pounds of flesh, improved in color and strength, and in power of resisting changes of weather, and ceased to have exacerbations of pain. He became able to walk with crutches, then with a cane, and before he left the hospital could walk unaided. The muscles of the limbs were developing satisfactorily, and there was every prospect of complete recovery.

I have alluded to the fact that in this instance the thorough use of mineral baths and waters had failed entirely to afford relief, but there are many cases where the proper use of these powerful agents gives us the best possible results. Judging from my own experience, I should say that the cases best adapted to their use are those where the system is not yet too far reduced, so that the power of reaction is not too feeble. It is indeed upon their power of developing reaction, and thus inducing more vigorous circulation and more healthful secretory activity of the skin, that mineral baths depend for their value in the treatment of rheumatism. It is evident, therefore, that they are to be regarded only as adjuvants, and that at the same time a most careful dietetic, hygienic and medicinal treatment must be carried out. It is largely owing to the universal neglect of this treatment at all American springs that such frequent disappointment awaits rheumatic patients who resort to them for relief. It is not difficult, however, to institute a suitable system of bathing at home, by which many of the good results of this important element in the treatment of chronic rheumatism may be obtained.

We have as yet but little positive knowledge in regard to the essential nature of rheumatism or of gout. All are agreed in regarding acute inflammatory rheumatism as a constitutional disease, although the widest diversity of opinion exists as to its true causes. So, too, there can be no doubt that in many cases of chronic rheumatism; there is the same constitutional disturbance which has assumed the chronic form, either from a repetition of acute attacks or from some peculiar modifying condition that has rendered it chronic from the outset. In such cases there probably exists some defect of primary assimilation or in the action of the great emunctories—liver, kidneys and skin. It is in consequence of this that in so many cases of true chronic rheumatism, great benefit is often derived from careful dietetics—such articles as close observation shows to be digested and assimilated with difficulty being restricted in amount or entirely prohibited. In whatever manner chronic rheumatism may have originated, nothing is more interesting and important than the part which the skin plays in keeping up the skin. So relaxed does the tone of the skin become under the influence of repeated attacks of acute rheumatism, or from unfavorable hygienic conditions, that, finally, the most trifling atmospheric changes, a momentary exposure to draught while the body is heated, or many other similarly slight causes, suffice to check the circulation and secretion of the skin and to induce an increase of rheumatic suffering. It would appear, therefore that any plan of treatment of chronic rheumatism which does not include a most careful attention to the state of the skin must, of necessity, fail in effecting a permanent cure ; and clinical experience thoroughly confirms this view. The requirements of individual cases must determine the precise character of this part of the treatment (whether by dry friction, inunction, cold or hot sponge-baths or douches, medicated baths, etc.); but it is sufficient now to request your attention to this as, perhaps, the most important element—though only one of several elements—in the truly curative treatment of chronic rheumatism.

I have thus far made scarcely any allusion to the large group of valuable remedies—mostly of an alterative character—that have acquired reputation in the treatment of chronic rheumatism.

It is true that in no case of this kind can we afford to depend solely on the use of any of these remedies, to the exclusion of baths, massage, diet, hygiene, it is no less true that in nearly every case there are indications that call for the use of some one or more of them. It would be impossible to discuss at length the merits of the very numerous remedies of this class, so that I must limit myself to the bare mention of those which have proved most valuable in my own experience.

In cases of chronic rheumatism limited to one or a few joints with considerable effusion, I have used the following with advantage:

℞

Potassii iodidi, ʒ ij.
Hydrargyri bichloridi, gr. j.
Syrup. sarsæ comp., ℥ v.
Ft. sol. S. Teaspoonful in water after meals.

or:

℞

Hydrargyri bichloridi, gr. j.
Inf. gentianæ comp., ℥ vij.
Ft. sol. S. 1 to 2 teaspoonfuls in water after meals three times daily.

In cases where a number of joints are involved with marked tendency to exacerbations, and especially if the lesions of the small joints indicate gouty complications:

℞

Pulv guaiaci, ʒ j.

Vin colchici radicis, ʒ ij. to ʒ iij.

Potassii iodidi, ʒ j.

Pulv acaciæ, q. s.

Sp. lavendulæ comp., ℥ ss.

Aq. cinnamomi, q. s. ad ℥ vj.

Ft. sol. S. Dessertspoonful three times daily in water.

The bicarbonate or the acetate of potash may often be substituted with advantage to the digestion for the iodide of potassium in the above mixture. I have already alluded to the use of prolonged courses of lithia as being very beneficial, especially in cases with a gouty element and with defective action of the kidneys. In regard to the mode of its administration, I much prefer the effervescing granulated salts.

I must also mention the benefit I have derived from the prolonged use of carefully increased doses of Donovan's solution. It is to be remembered that these alteratives have, for the most part, been given while the patient was also taking iron in large doses, cod liver oil, syr. hypophos. comp., or some similar nutrient.

I will merely mention again the nitrate of silver as an alterative, from which I think I have obtained good results, especially in cases attended with neuritis and with marked nervous symptoms.

